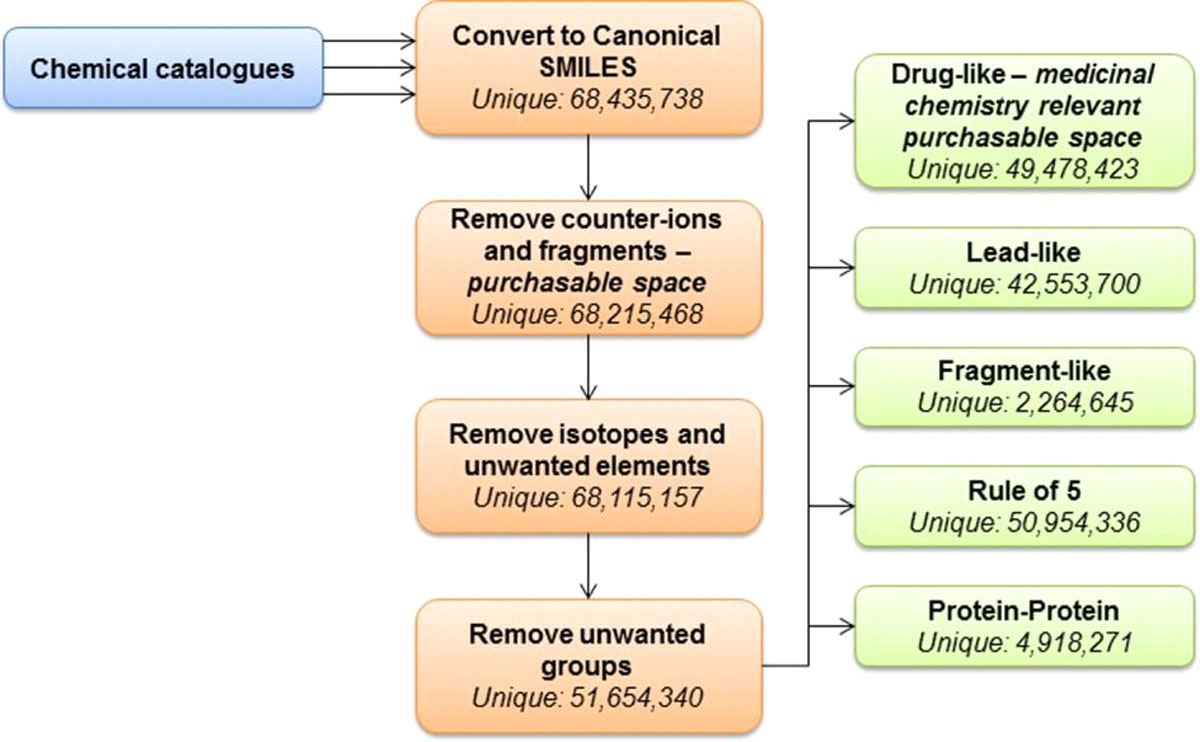# ChemicalToolBoX and its application on the study of the drug like and purchasable space

**DOI:** 10.1186/1758-2946-6-S1-P51

**Published:** 2014-03-11

**Authors:** Xavier Lucas, Björn A Grüning, Stefan Günther

**Affiliations:** 1 Pharmaceutical Bioinformatics, Institute of Pharmaceutical Sciences, Albert-Ludwigs-University, Freiburg, D-79104, Germany

## 

The ever increasing amount of data and computational capabilities in the cheminformatics field has led to a scenario where efficient techniques for storage and processing in an integrated, modular, and easily accessible platform are in vital demand. Here, we present ChemicalToolBoX, a compilation of more than 30 tools integrated into a single computational chemistry and cheminformatics platform based on the Galaxy workflow management system [1,2]. We have recently designed a workflow within the ChemicalToolBoX to generate a library of compounds containing around 70 million unique commercially available small molecules, i.e. the purchasable space [3]. Subsequently, we have used filtering rules based on structural patterns and chemical alarms to discard problematic molecules, representing a very large portion of the drug-like and purchasable space, along with other drug discovery data sets including more than 2 million fragments (Figure 1). Furthermore, we have computed several physicochemical descriptors to discover general trends applying to each subset.

**Figure 1:**